# A Review of the Early Detection of Colon Cancer and the Role of Circulating Tumor DNA

**DOI:** 10.7759/cureus.84394

**Published:** 2025-05-19

**Authors:** Arjun Kandel, Aye M Thida, Mohan Preet

**Affiliations:** 1 Internal Medicine, State University of New York Downstate Medical Center, Brooklyn, USA; 2 Hematology and Oncology, State University of New York Downstate Health Sciences University, Brooklyn, USA; 3 Hematology and Oncology, State University of New York Downstate Medical Center, Brooklyn, USA

**Keywords:** ca colon, cancer early detection, colorectal cancer, ctdna, liquid biopsy

## Abstract

Colorectal cancer (CRC) is the third most commonly diagnosed cancer and the second leading cause of cancer-related deaths when both males and females are considered. Screening plays a crucial role in reducing the incidence and mortality of CRC. Traditionally, CRC screening methods are classified into stool-based and visual-based approaches. Stool-based tests include the guaiac fecal occult blood test, fecal immunochemical test, and multitarget stool DNA test, while visual-based methods encompass colonoscopy, flexible sigmoidoscopy, CT colonography, and capsule endoscopy. Circulating tumor DNA (ctDNA) has emerged as a promising, minimally invasive tool, offering encouraging sensitivity and specificity. However, its high cost remains a limitation. In this review, the authors present a comprehensive overview of current CRC screening tools, with a particular focus on the role of ctDNA.

## Introduction and background

Globally, colorectal cancer (CRC) accounts for approximately 10% of all diagnosed cancer cases. It is the third most commonly diagnosed cancer, following breast and lung cancer in females and lung and prostate cancer in males. In 2022, an estimated 1.93 million new CRC cases were reported worldwide, including one million cases in males and 0.9 million in females. The disease also caused approximately 904,019 deaths globally: around 500,000 in males and 400,000 in females [1-4].

In the US, there were an estimated 153,020 new CRC cases, with 106,970 affecting the colon and 46,050 the rectum. Of these, 81,860 occurred in males and 71,160 in females. CRC was also responsible for about 52,550 deaths: 28,080 in males and 24,080 in females [5,6].

Various organizations have introduced multiple screening options to expand patient choices and increase participation in screening programs [7]. CRC screening is conventionally divided into two categories: stool-based tests and visual inspection methods, which include both endoscopic and imaging techniques. Stool-based tests detect heme, hemoglobin, or molecular biomarkers in feces. These include high-sensitivity guaiac fecal occult blood tests (gFOBTs), fecal immunochemical tests (FITs), and stool DNA testing. Most global screening programs rely heavily on FOBT [8]. Visual inspection methods include colonoscopy, flexible sigmoidoscopy (FS), and colon capsule endoscopy (CE) [9].

The inclusion of colonoscopy in Medicare coverage in the US significantly increased its use [8]. In 2018, 61% of individuals aged 50 and older reported using colonoscopy for CRC screening [6]. In addition to conventional techniques, circulating tumor DNA (ctDNA) has emerged as a promising biomarker. It offers valuable information for diagnosis, prognosis, and monitoring of recurrence before, during, and after CRC treatment [10,11].

Early detection is key to reducing CRC-related mortality. Previous studies have consistently shown that early detection correlates with a decrease in both incidence and death rates from CRC [12-14]. A systematic review and meta-analysis by Zheng et al. found that screening significantly reduces CRC mortality. The study included 10 randomized controlled trials (RCTs) and 47 modeling studies. Among the RCTs, the mortality rate ratio for gFOBT was 0.88 (95% CI: 0.80-0.96), and for single FS, it was 0.76 (95% CI: 0.68-0.84). Model-based estimates showed mortality rate ratios of 0.45 (95% CI: 0.39-0.51) for biennial FIT, 0.61 (95% CI: 0.53-0.72) for single FS, 0.27 (95% CI: 0.21-0.35) for colonoscopy, 0.31 (95% CI: 0.28-0.34) for gFOBT, and 0.35 (95% CI: 0.29-0.42) for FS performed every five years [12].

A large community-based nested case-control study by Doubeni et al., which included 1,747 CRC mortality cases and 3,460 controls without CRC, reported that colonoscopy screening reduced CRC mortality risk by 67% (adjusted OR = 0.33; 95% CI: 0.21-0.52) [13]. Similarly, a prospective study by Schoen et al. involving 154,900 participants - half assigned to a screening group and half to usual care - found a 21% reduction in CRC incidence (11.9 vs. 15.2 cases per 10,000 person-years) and a 26% reduction in CRC-related deaths (2.9 vs. 3.9 deaths per 10,000 person-years) in the screened group [14].

This review examines the strengths and limitations of current CRC screening tools and explores the emerging role of ctDNA in the diagnosis and prognosis of CRC patients.

## Review

Methodology

A search was conducted in the PubMed database from January 1, 2024 to April 30, 2025 using Medical Subject Headings (MeSH) terms and free-text keywords within abstracts. All searches employed Boolean logic, combining search terms with the operators "AND" and "OR." The search strategy was developed using the following keywords: “Colorectal cancer”, “Circulating tumor DNA”, and “Early detection of cancer”.

Current screening tests for CRC in the US

CRC screening guidelines currently vary across different organizations in the United States. Key organizations - including the American Cancer Society, American College of Gastroenterology, United States Preventive Services Task Force, United States Multi-Society Task Force, American College of Physicians, and National Comprehensive Cancer Network - primarily recommend stool-based tests and visual inspection methods. These include FIT, gFOBT, multitarget stool DNA test (mt-sDNA), colonoscopy, computed tomographic colonography (CTC), FS, and colon video CE.

Most of these organizations recommend initiating screening between the ages of 45 and 50, with a strong consensus to begin routine screening from age 50 to 75. The shift toward earlier screening may be linked to the rising incidence of CRC in younger populations [15]. Screening is generally not recommended beyond the age of 85. The recommended intervals for various screening tests are outlined in Table 1 [16-21].

**Table 1:** Recommended screening intervals for CRC tests by various US organizations Although screening intervals vary slightly among guidelines, all organizations recommend regular screening for adults aged 45 to 75 years. ACP, American College of Physicians; ACG, American College of Gastroenterology; ACS, American Cancer Society; CE, capsule endoscopy; CRC, colorectal cancer; CTC, computed tomographic colonography; FIT, fecal immunochemical test; FS, flexible sigmoidoscopy; gFOBT, guaiac fecal occult blood test; mt-sDNA, multitarget stool DNA; NCCN, National Comprehensive Cancer Network; USPSTF, United States Preventive Services Task Force; USMSTF, United States Multi-Society Task Force

Screening test	ACS (2018) [16]	ACG (2021) [17]	USPSTF (2021) [18]	USMSTF (2017) [19]	ACP (2020) [20]	NCCN (2021) [21]
FIT	One year	One year	One year	One year	Two years	One year
gFOBT	One year	One year	One year	Not included	Two years	One year
mt-sDNA	Three years (manufacturer dependent)	Three years	One to three years	Three years	Not included	Three years
Colonoscopy	10 years	10 years	10 years	10 years	10 years	10 years
CTC	Five years	Five years	Five years	Not included	Five years	Five years
FS	Five years	Five to 10 years	Five years	Five to 10 years	10 years	Five to 10 years
CE	Not included	Five years	Not included	Five years	Not included	Not included

Stool-based screening tests

gFOBT

The gFOBT detects heme in stool through peroxidase activity to help diagnose CRC [22]. It does not require gastrointestinal preparation and aids in early CRC detection. However, it requires dietary restrictions and the collection of three stool samples. The test has relatively high rates of false positives and false negatives, with one false positive in 82 cases and one false negative in 182 cases, which may lead to unnecessary colonoscopies or missed cancers [20]. A meta-analysis of 26 studies reported a sensitivity of 0.68 and specificity of 0.88 for gFOBT [23]. While gFOBT is sensitive to lower gastrointestinal bleeding, it may detect bleeding from the entire GI tract because heme is relatively stable along the alimentary canal [22]. Nur et al. found that screening for CRC using gFOBT combined with colonoscopy or sigmoidoscopy yielded quality-adjusted life years (QALYs) of 7.7 at a cost of 3,573 USD, compared to no screening with a QALY of 7.2 and a cost of 4,084 USD. Screening using only sigmoidoscopy or colonoscopy had QALYs of 6.8, with costs of 4,905 USD and 5,002 USD, respectively, indicating that FOBT with follow-up endoscopy offers a cost-effective screening method [24].

FIT

FIT detects intact hemoglobin in stool to diagnose CRC [25]. Because globin is rapidly digested in the upper GI tract, FIT results are specific to the colorectal region [22]. FIT benefits patients as it requires no gastrointestinal preparation, uses only a single stool sample, has fewer than one false negative per thousand tests, and does not require dietary restrictions. However, approximately 1 in 11 tests may lead to unnecessary colonoscopy [20,26]. Compared to gFOBT, FIT offers higher sensitivity and specificity and requires only one stool sample instead of three [11,26]. A 2019 meta-analysis of 32 studies reported FIT sensitivity for CRC at 0.91 (at 10 µg/g) and 0.75 (at 20 µg/g), with specificities of 0.90 and 0.95, respectively. For advanced adenomas, sensitivities were 0.40 and 0.25 at these thresholds [27]. Although FIT may have higher sensitivity than gFOBT, both tests have relatively low sensitivity overall [28]. Zhong et al. reported better participation and cost-effectiveness for FIT compared to colonoscopy every 10 years, although adenoma detection rates were lower for FIT [29].

mt-sDNA

mt-sDNA detects multiple biomarkers in stool associated with polyps and CRC. It requires no dietary restrictions and can detect neoplasms at multiple sites. However, the test is costly, the recommended screening interval of three years limits long-term data, and mt-sDNA has lower sensitivity for advanced precancerous lesions [30]. A cross-sectional study of 9,989 participants found mt-sDNA sensitivity for CRC at 92.3%, but sensitivity dropped to 42.4% for advanced precancerous lesions; specificity was 86.6% [31]. Redwood et al. reported that perfect adherence to mt-sDNA screening extended life by 0.19 QALYs per person, compared to 0.17 for FIT and 0.15 for colonoscopy. With imperfect adherence, mt-sDNA extended life by 0.12 QALYs per person, while FIT and colonoscopy extended life by 0.05 and 0.06 QALYs, respectively. mt-sDNA also preserved more QALYs at a lower cost than FIT [32].

Visual-based screening tests

Colonoscopy

Colonoscopy is an endoscopic procedure that screens the entire colorectal lining [33]. It allows detection and removal of precancerous lesions and early-stage cancers during the procedure [34]. However, it is invasive, expensive, requires bowel preparation, and carries risks such as perforation and hemorrhage. Psychological and financial barriers contribute to low adherence rates, reported between 10% and 27% [11]. Lin et al. summarized data from three studies showing adenoma sensitivity between 0.75 and 0.93 for lesions ≥6 mm, with specificity around 0.94 (although only one study reported this). For adenomas ≥10 mm, sensitivity ranged from 0.89 to 0.95, with specificity at 0.89 [35].

FS

FS is similar to a colonoscopy but examines only the rectum and distal colon. Like colonoscopy, it requires no sedation and can be performed outpatient, with abnormal findings referred for colonoscopy. FS carries lower risks of perforation and hemorrhage compared to colonoscopy [36]. FS preparation with an enema alone is effective, and patient compliance tends to be better than for colonoscopy. The procedure is shorter and less costly [37]. A 2017 meta-analysis reported FS sensitivity for CRC and advanced adenoma at 67% (range 59-79%) [38].

CTC

CTC uses multirow detector CT to generate high-resolution images of the large intestine, aiding lesion detection, characterization, and localization. It requires extensive bowel preparation and colonic gas distension; residual fecal matter may cause false positives [39]. A 2020 meta-analysis found CTC sensitivity of 0.87 and specificity of 0.90 for polyps ≥6 mm and sensitivity of 0.91 and specificity of 0.98 for polyps ≥10 mm [40].

CE

CE visualizes the colonic mucosa without gas insufflation and requires no sedation. It offers an advantage over CTC by providing direct visualization of the colon [41]. Although convenient and less invasive, CE captures passive images and may miss lesions. Secretions in the alimentary canal can obstruct views, the procedure is time-consuming, and biopsy samples cannot be obtained [42]. A 2022 meta-analysis reported CE sensitivity of 88% for polyps ≤6 mm and ≤10 mm, with specificities of 94% and 95.5%, respectively [43].

The characteristics of stool-based and visual-based screening tests for CRC are presented in Table 2.

**Table 2:** Characteristics of stool-based and visual-based screening tests for CRC CE, capsule endoscopy; CRC, colorectal cancer; CTC, computed tomographic colonography; FIT, fecal immunochemical test; FS, flexible sigmoidoscopy; gFOBT, guaiac fecal occult blood test; mt-sDNA, multitarget stool DNA

Screening test	Stool based			Visual based			
	gFOBT	FIT	mt-sDNA	Colonoscopy	FS	CTC	CE
Mechanism	Chemical guaiac reaction	Antibody-antigen detection	Identifies biomarkers	Endoscopic technique	Endoscopic technique	CT	Capsule with camera
Strengths	Noninvasive	Noninvasive; single sample; no diet restriction	Noninvasive; detects from multiple sites; no diet restriction	Detection and possible removal during the procedure	No sedation required	Noninvasive	Less invasive; no gas distention; direct visualization of the colon
Limitations	Requires multiple samples; diet restrictions	Detects only in the colorectal region	High cost	Invasive; high cost; bowel prep required; risk of complications	Limited to the distal colon	Requires bowel prep and gas distention	Passive images; obstructed images due to secretions; long duration
Specificity	0.88	0.91	0.86	0.75-0.93	0.59-0.79	0.87	0.88
Sensitivity	0.68	0.75	0.92	0.94	-	0.9	0.94

ctDNA: an emerging screening technique for CRC

Liquid biopsy (LB) enables molecular analysis of body fluids, broadening research possibilities and deepening our understanding of malignancies. Various fluids such as blood, urine, ascites, pleural fluid, and cerebrospinal fluid can be used to isolate circulating extracellular nucleic acids, including cell-free DNA and ctDNA. Tumor-derived cells, primarily found in blood, circulate briefly, typically for one to two and a half hours, and can be detected using techniques like real-time quantitative PCR, digital droplet PCR, and next-generation sequencing. These biopsies offer advantages including lower cost, the ability to detect tumors from multiple sites, and the possibility of obtaining serial samples for screening and monitoring treatment response. However, challenges remain, such as the low concentration of tumor DNA in blood, variability in reproducibility across different manufacturers, early acquisition of hematological mutations, and other technical limitations [44].

ctDNA is gaining attention as a promising tool for CRC screening, prognosis evaluation, treatment monitoring, and guiding therapy. Detection approaches include targeted techniques, which look for known genetic mutations, and untargeted methods that do not require prior genetic knowledge [13]. Detection rates of ctDNA in localized lesions vary widely across studies, ranging from 40% to 100%, while detection in metastatic disease reaches nearly 100% [13,45,46].

The sensitivity and specificity of ctDNA reported in previous studies vary depending on the study design and the techniques employed, as summarized in Table 3. Sensitivity values as high as 93% and specificity up to 92% have been observed in the literature [47-49]. As noted earlier, studies show that FIT, mt-sDNA, colonoscopy, CTC, and ctDNA all demonstrate sensitivities greater than 90% for CRC. Similarly, FIT, colonoscopy, CTC, CE, and ctDNA exhibit specificities exceeding 90%. However, to draw definitive conclusions, larger sample sizes are needed to better characterize the sensitivity and specificity of ctDNA.

**Table 3:** Sensitivity and specificity of ctDNA for CRC Sensitivity and specificity reported in previous studies vary depending on the study design and the techniques used. bDNA, branched DNA assay; ctDNA, circulating tumor DNA; ddPCR, droplet digital PCR; MSP, methylation-specific PCR; RT-qPCR, quantitative reverse transcription PCR

Study	Sample size	Study design	ctDNA technique	Sensitivity	Specificity
Bessa et al. (2023) [47]	623	Cohort	Next-generation sequencing	93%	90%
Brenne et al. (2023) [48]	212	Case-control	Targeted PCR	43%	86%
Min et al. (2023) [49]	25,240	Meta-analysis	RT-qPCR, ddPCR, bDNA, fluorescent dye assay, MSP	73%	92%

Adler et al. found that only 37% of participants complied with colonoscopy screening. Among those who refused, 83% preferred the Septin9 blood test as an alternative [50]. Likewise, Osborne et al. reported that 78% of participants favored a blood test over a stool test, likely due to perceived comfort, acceptability, and convenience [51].

Aziz et al. showed that hybrid strategies combining colonoscopy and LB resulted in the greatest life years gained; however, for LB to be cost-effective, its cost would need to drop by 66%. Compared to no screening, the cost would have to decrease by 94% to meet the incremental cost-effectiveness threshold. For stool-based tests, cost reductions between 43% and 80% were required to achieve cost-effectiveness [52]. In contrast, Schneider et al. reported that adding ctDNA analysis via molecular residual disease assays to traditional high-risk clinicopathological predictors increased life years by 0.8 and QALYs by 0.7 per patient while saving $9,771 per patient. They concluded that incorporating ctDNA improves treatment decisions for resectable CRC patients undergoing chemotherapy [53].

Strengths and Limitations of ctDNA

Sampling for ctDNA is minimally invasive, even for tumors difficult to access with traditional biopsies [54]. Although tissue biopsy remains the gold standard for molecular profiling, it may be associated with more complications, inadequate sample acquisition, and longer turnaround times compared to ctDNA. This makes ctDNA particularly suitable for repeated serial assessments [55-57]. CtDNA also predicts prognosis in treated patients. Reinert et al. reported that 14 of 16 CRC patients positive for ctDNA after definitive therapy relapsed; all seven ctDNA-positive patients following adjuvant chemotherapy relapsed. Post-treatment, ctDNA-positive patients had a 40-fold higher risk of relapse than ctDNA-negative patients [58].

Targeted therapy selection can benefit from ctDNA, as it provides genetic profiles of both primary and distant lesions [59]. CtDNA profiling is useful in managing tumor heterogeneity over time [56]. For instance, patients treated with anti-EGFR antibodies for RAS mutations may develop resistance due to mutations detectable by ctDNA, enabling real-time monitoring of such changes [60]. Kumar et al. described an 81-year-old woman whose tissue NGS showed microsatellite stability, but ctDNA NGS revealed high microsatellite instability, mismatch repair deficiency, and BRAF mutation, significantly altering her treatment and prognosis [61]. Moreover, ctDNA use has demonstrated cost savings for Medicare Advantage and commercial payers [62].

However, ctDNA faces challenges, including reduced sensitivity for detecting metastases in sites like the peritoneum and lungs, where tumor DNA shedding is lower [59]. Early-stage CRC detection is limited by low biomarker quantity [63]. Since ctDNA derives from apoptotic or necrotic tumor cells, smaller tumors shedding fewer cells are harder to detect [64]. Detection requires prior knowledge of target mutations, although multiplex assays can screen for limited known mutations [65]. CtDNA has a short half-life (less than two and a half hours), and discordance with tumor tissue testing may occur, which should be considered in clinical interpretation [66]. Furthermore, LB costs currently exceed many willingness-to-pay thresholds [52].

Recommendations

CtDNA is a promising tool for CRC screening, prognosis prediction, and treatment monitoring. While previous studies report encouraging sensitivity and specificity, larger prospective studies are essential to confirm these metrics. Patient compliance with CRC screening may improve with less invasive and more convenient alternatives like ctDNA. Nonetheless, strategies to reduce ctDNA costs are necessary for broader implementation.

## Conclusions

CtDNA holds great potential in early CRC detection, offering a minimally invasive option with fewer complications than traditional biopsy, which could enhance patient compliance. It provides comprehensive tumor profiling from both primary and metastatic sites and facilitates real-time monitoring of tumor mutations during therapy. Despite current limitations such as high costs, variable sensitivity, and limited data, overcoming these challenges may justify including ctDNA in future CRC screening guidelines.
